# Nanoglasses: a trail towards uncharted regions of vitreous materials and their properties?

**DOI:** 10.3762/bjnano.17.72

**Published:** 2026-08-07

**Authors:** Gerhard Wilde, Horst Hahn

**Affiliations:** 1 University of Münster, Institute of Materials Physics, 48149 Münster, Germany; 2 Herbert Gleiter International Institute, Liaoning Academy of Materials, Shenyang 110167, P. R. China; 3 Department of Materials Science and Engineering, University of Arizona, 1235 James E. Rogers Way Tucson, AZ 85719, United States; 4 Institute of Nanotechnology, Karlsruhe Institute of Technology, Kaiserstr. 12, 76131 Karlsruhe, Germany

**Keywords:** columnar thin-film nanoglasses, glass–glass interfaces, mechanical and functional properties, nanoglass, stability

## Abstract

Nanoglasses are a class of non-crystalline materials composed of nanoscale glass regions separated by glass–glass interfaces that possess distinct structural and thermodynamic characteristics. These internal interface regions with finite width of the order of several nanometres introduce excess free volume, altered short- and medium-range order, including the distribution of chemical constituents, and modified energetic states compared with conventional homogeneous glasses, giving rise to unique structural and functional properties. Additionally, the interfaces between the glass cores and the glass–glass interfaces need to be considered, as the specific interface areas are large. In this perspective paper, we discuss the structure, thermodynamics, and stability of glass–glass interfaces and examine their implications for the design and properties of nanoglasses, highlighting that this approach represents a novel pathway for modifying amorphous materials more broadly. Particular attention is given to columnar thin-film nanoglasses, where vertically aligned glassy columns create a high density of internal interfaces. We outline how interfacial excess energy, configurational entropy, and relaxation processes influence the metastability and properties of these materials. Furthermore, we discuss perspectives on how the control of interface density and chemistry may enable tailoring of mechanical, diffusion, and functional properties, including enhanced plasticity, altered transport behaviour, and tuneable optical or electronic responses. Finally, open questions regarding interfacial structure, thermodynamic driving forces, and stability are identified, emphasizing opportunities for integrating nanoglasses into advanced functional thin-film systems.

## Introduction

The advancement of civilization has always been closely tied to the continuous development of new and improved materials. From the earliest stages of material use, humans learned to modify the structure and composition of materials to enhance their performance and expand their applications. While chemical modifications play a key role, though they are not considered here as the thermodynamics of heterogeneous systems is a well-established field, mechanical processing and heat treatment have been central to the engineering of microstructures with ever-increasing precision. Over time, this has progressed to controlling structures at the nanometre-level and even at the atomic scale. Despite these widespread concepts of modification of materials, materials science has traditionally focused on crystalline materials, particularly in the context of microstructure and defect engineering, including the manipulation of defects such as internal interfaces and dislocations. In contrast, amorphous materials such as metallic glasses have largely been overlooked in the aspect of microstructure and defect engineering.

Yet, glasses are ubiquitous in both our daily life and in nature, and they have accompanied humanity’s technological and societal development since the Stone Age. One of the most significant distinctions between glasses and crystalline materials is the ability to tune their properties through processes such as aging, relaxation, or rejuvenation. In fact, glasses offer a unique opportunity to modify their properties by altering the specific volume and/or the local atomic structure via various processes, including deformation, irradiation, or heat treatment. The magnitude of the changes that can be permanently induced in glasses far exceeds the corresponding modifications achievable in crystalline material of similar composition. This difference arises from the distinct atomic structures of crystalline or vitreous materials, where the rigidity of the crystalline order restricts significant changes in specific volume or local atomic arrangements within a given crystal structure and for the same chemical constituents. Historically, the “structural flexibility” of glasses has primarily been harnessed in response to thermal treatments. These modifications are often described in a mean-field approach, attributing the changes in properties to variations in the average excess volume that is often termed “free volume” [1–2]. However, the reliance on thermal processing limits the range of excess volume changes to only 1–2%. One should keep in mind that this range of excess volume variation is still much larger for glasses compared to crystalline materials. When limiting the discussion to point defects, namely, vacancies, as the principal contributors to excess volume in crystalline materials, one typically observes concentrations of the order of 10^−4^ near the melting point. These concentrations, corresponding to approximately 0.1% excess volume, exhibit an exponential decline with decreasing temperature. Thus, variations of the number density of point defects in crystalline materials within the documented range correspond to minor variations in the excess volume. Yet, also for glasses and with the absence of a crystalline lattice and the restrictions concerning excess volume that this poses, this range of 1–2% is a relatively narrow perspective on property modification, especially when compared to techniques in crystalline materials like grain refinement or work hardening, which can lead to much more significant changes in properties and which are not bound to point defects but to grain boundaries or dislocations as defects of the crystal lattice that have no correspondence in uniform glassy materials.

Also, it is now widely accepted that the local (atomic-scale and nanometre-scale) structure in a glass changes too when its excess volume is modified. However, due to the complexities involved in characterizing and describing disordered structures, changes in local atomic arrangements have rarely been quantified, and this field is currently still at an early stage. Nevertheless, the relationship between structure and properties, even for disordered materials like glasses, raises intriguing questions: Can glasses with a given chemical composition undergo “microstructural” engineering beyond the well-known and limited changes in specific volume? Can we impose a tailored “microstructure” onto glasses to control their properties? This line of inquiry extends further, exploring the potential extent of structural modifications and correlated property changes. It also ventures into a more philosophical territory, pondering the possible existence and nature of structural defects within a disordered, vitreous solid and also considering the theoretically lowest free enthalpy state of a “defect-containing” glass.

Although the original concept dates back to the 1980s [3], it is only recently that “nanoglasses” have gained international attention as a new approach to tailoring glass properties. With advances in synthesis and processing techniques for nanoscale systems, nanoglasses have transitioned from theoretical concepts to real-world materials [4–6]. Nanoglasses consist of vitreous cores (or “glass grains”) connected by amorphous regions, referred to as “glass–glass interfaces (GGIs)”, which exhibit structures and properties that go beyond those achievable in conventional, structurally uniform glasses. The glass–glass interfaces are characterized as regions several nanometres in thickness, exhibiting excess volumes that can substantially exceed those attainable through conventional liquid quenching, aging, or rejuvenation with reported values up to an order of magnitude higher [7–8]. It is important to emphasize that the combination of properties found in glasses with such glass–glass interfaces distinguishes them from all known interfaces in crystalline materials. Given the correlation between excess volume and interatomic interaction potential, significant changes in nearly all material properties are expected. This new pathway for the creation of “glasses with a microstructure”, that is, nanoglasses, thus opens up exciting opportunities for the engineering and tailoring of amorphous materials through nanostructuring, potentially also advancing our understanding and application of vitreous materials in general.

**Figure 1:**
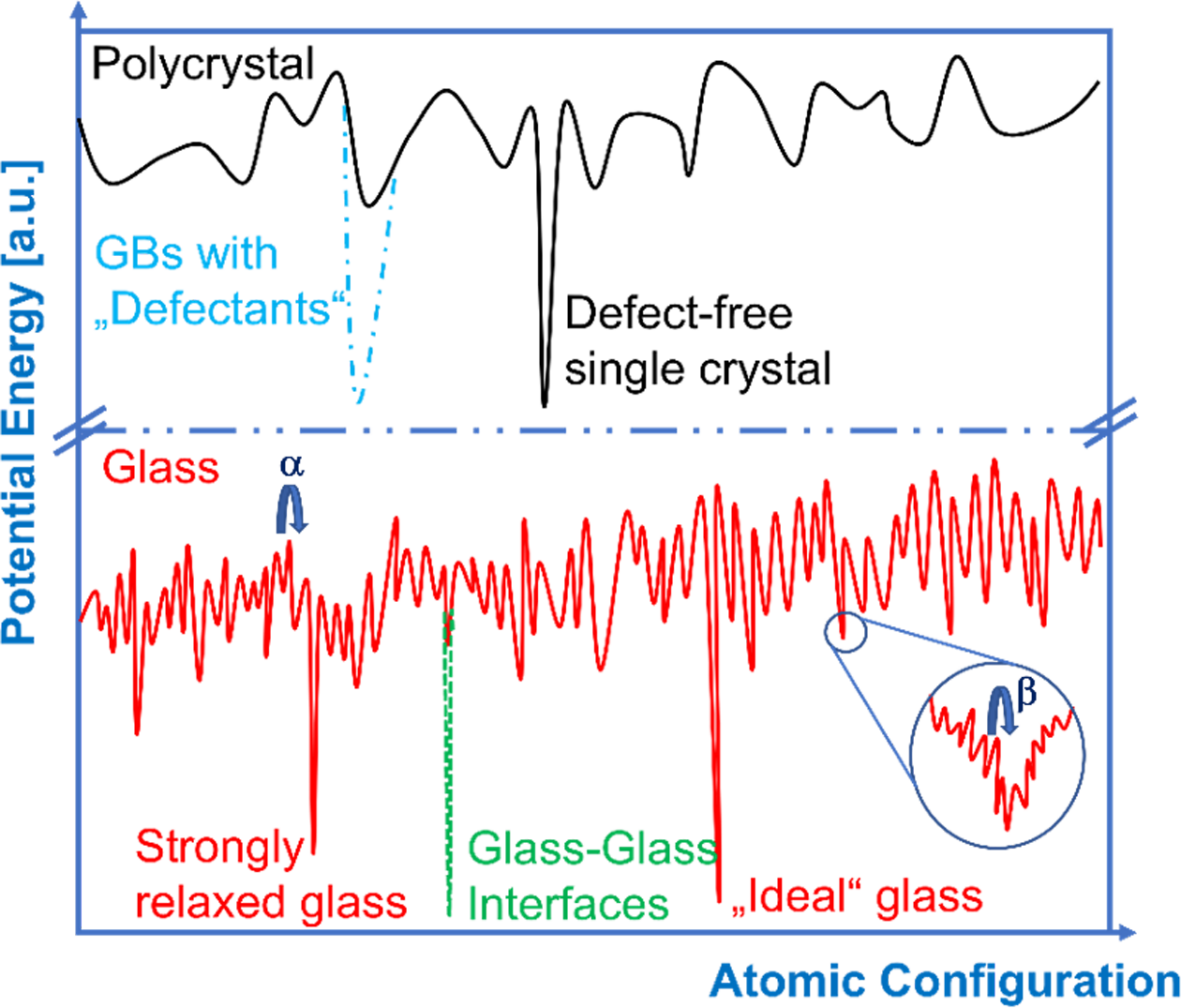
Comparison between the energy landscapes of polycrystal and glass states. Note that a direct comparison between the hypothetical ideal glass state and the ideal single crystal is meaningless. Thus, the two energy landscapes are plotted on individual axes. Multicomponent materials are taken into account as no nanoglass with only one constituent is known. Thus, also the polycrystal state has various configurations, given, for example, by different segregation behaviour to one-, two-, or three-dimensional defects. Hashed light blue indicates the “defectant” concept as suggested by [16]. According to this defectant concept, the minimal potential energy of defected grain boundaries might be similar to the potential energy of the defect-free single crystal. Even lower values of the grain boundary excess energy density would cause spontaneous grain refinement, which so far has not been reported. The larger width of the minimum associated with grain boundaries corresponds to the spectrum of grain boundary structures. For a glassy material (of the same hypothetical composition as for the polycrystal), a much larger number of possible metastable configurations exist. The energy landscape indicated in red also contains a much finer structure, leading to the well-known relaxation behaviour of glasses. In metallic glasses, the characteristic energy scales for the distinct α-, β- and γ-relaxation processes are usually discussed in terms of activation energies. The exact values depend strongly on alloy composition, fictive temperature, pressure, and measurement method, but the robust order-of-magnitude ranges are about 2–5 eV per event for α-, about 0.7–1.5 eV per event for β-, and about 0.05–0.5 eV per event for γ-relaxations. γ-Relaxations have not been visualized in the figure as they would appear too small. Nanoglass interfaces have been reported to reveal a “unique” structure for a given composition. Thus, the width of the associated minimum in the energy landscape is narrower.

Nanoglasses introduce structural heterogeneity and an additional characteristic length scale into fully amorphous materials. Due to the large variety of local structures, or “motifs”, in glasses, reflected in the complex energy landscapes of vitreous materials (see below in Figure 1), the potential structures can vary much more significantly than in any crystalline state. In fact, it is only through the consideration of processing routes beyond the conventional liquid quenching and subsequent relaxation methods, such as gas-phase condensation, sputtering, or electrochemical deposition, that new structural motifs in previously unexplored regions of the complex energy landscape of glasses may become accessible. One recent example is given by so-called “ultrastable glasses”, where specific conditions for the formation during deposition of thin-film glasses have led to the discovery of relaxation states hitherto thought of as inaccessible, even on extended time scales surpassing the duration of humanity’s era of materials use [9].

This article presents new perspectives on nanoglasses, with particular emphasis on their thermodynamic description, the observed stability of these materials, their range of achievable structural variations and the distinctive set of glass properties that may result. It further outlines promising directions for future research.

## Perspective

### Vitreous and amorphous solids: materials with complex energy landscapes

The structure of amorphous materials, such as glasses, is often described by the term “disorder”. Yet, it is the absence of long-range periodic order that distinguishes the structure of this class of materials from crystalline solids. On more local scales, amorphous materials possess distinct structural arrangements that have been termed as short-range order (SRO) [10] and medium-range order (MRO) [11]. In that context, SRO describes pair correlations extending over the first few nearest-neighbour shells and is an immediate result of the interatomic interaction potentials. MRO has received different definitions in the literature, yet the most accepted view is that of higher-order (e.g., pair–pair) correlations in space [12]. The length scale over which MRO exists in glasses is of the order of a few nanometres, and any particular MRO is often referred to as “motif”. Given the absence of long-range periodicity, disordered structures consist of more than one motif, and many different models describing the intrinsically heterogeneous structure of glasses have been proposed. In essence, these models describe the absence of long-range periodicity by assembling different “motifs” in a non-periodic manner, allowing also for non-uniform excess volume distributions.

However, common to all structural descriptions of glasses is the uniformity of the structure on larger length scales. In fact, according to structural models and experimental observations, the structure of amorphous solids is uniform on a length scale of a few tens of nanometres in the absence of external forces or fields. Relaxation, aging, or rejuvenation change the local structure, that is, the distribution and arrangement of motifs and the distribution and amount of excess volume [13]. With the multitude of possible arrangements, a complex energy landscape results [14], which is schematically depicted in Figure 1. As discussed in detail in many reviews on glasses, the energy landscape exhibits a characteristic topology that includes so-called “meta-basins” and local minima on at least two different energy scales, which describe the relaxation states of glasses and their associated relaxation modes, namely alpha (α)-, beta (β)-, and gamma (γ)-relaxations [15] (Figure 1). Little is currently known about how the distribution and arrangement of local motifs correlate with relaxation, aging, or rejuvenation. Most models restrict their explanation of these processes to changes in the amount and/or spatial distribution of excess volume and to the presence of more or less “unfavourable” motifs.

With the advent of nanoglasses, a new spatial scale of structural heterogeneity was introduced, which is defined by the diameter of the “glass grains” and typically amounts to few tens of nanometres. Given a thickness of the glass–glass interfaces of the order of a few nanometres, it is expected that different structural arrangements exist within the grains in comparison to the glass–glass interfaces; this has been confirmed recently by so-called fluctuation electron microscopy [17]. Given the exceptionally large excess volume reported for the glass–glass interfaces, a distinct offset of the position inside an energy landscape model between the states of the glass grains and the glass–glass interfaces is expected. Additionally, the large amount of excess volume in the glass–glass interfaces might allow, in principle, for even more structural variability. Thus, an energy landscape of a nanoglass might bear similarity with the schematic indicated in the lower part of Figure 1. As indicated therein and in the corresponding figure caption, new relaxation states exist for the glass–glass interfaces. In addition, new meta-basins are expected since long-time stability of nanoglasses with grains and interfaces has been observed experimentally. In a simplistic picture, the glass grains and the GGI thus reside in different meta-basins of the energy landscape.

Yet, the situation might actually be even more interesting, due to the larger variability of structural motifs and (related) possible glass relaxations at increased excess volume. Additionally, a larger structural variability corresponds to a larger complexion number, that is, a larger configurational entropy, stabilizing this state by reducing the total free enthalpy, similar to what is intensively discussed for high-entropy alloys. Thus, due to these different reasons, structural states might exist in glass–glass interfaces that have a lower free enthalpy as compared to accessible states of the glass grains. This situation is also indicated in Figure 1. Similar arguments, that is, more efficient sampling of the energy landscape under specific deposition conditions, are underlying the initial discovery of ultrastable glasses [9], as also discussed in the context of nanoglasses [18–20]. In fact, in both cases it is the accessibility probability of states that are lying low in the energy landscape that constitute the reasoning for attaining glasses with new states. Yet, so far, the discussion of ultrastable glasses has mostly focused on accessing states within one given meta-basin (i.e., identical for melt-quenched or deposited glasses) while nanoglasses include necessarily structural heterogeneity, the glass–glass interfaces, which might reside in a different meta-basin, as indicated by the absence of any observation of GGIs in melt-quenched glasses.

An interesting question pertains to the “ideal glass” that hypothetically exists after infinitely slow cooling of a liquid while avoiding crystallization. Such an ideal glass state would have the same entropy (at the “Kauzmann temperature” [21] where the temperature-dependent entropies become equal) as an equilibrium crystal of identical composition. As a nanoglass, and particularly the material residing in glass–glass interfaces, is not confined to the configuration space accessible to glasses made by cooling a liquid through its glass transition, there is no general rule that confines its excess entropy to the same range as for melt-quenched glasses. However, similar to the classical argument by Kauzmann, it is difficult to imagine that the entropy of a disordered solid would be less than the entropy of a perfect single crystal of identical composition at some finite temperature and still obey the second law of thermodynamics as this would require distinct courses of the specific heats of both states down to the low-temperature limit. For this reason, Figure 1 displays the free enthalpy minimum for the GGI as a dashed curve with a minimum at equal level as the ideal glass. In this sense, the Kauzmann temperature is taken here as a material-inherent quantity that is determined by the interatomic interactions that also define the crystalline ground state.

**Figure 2:**
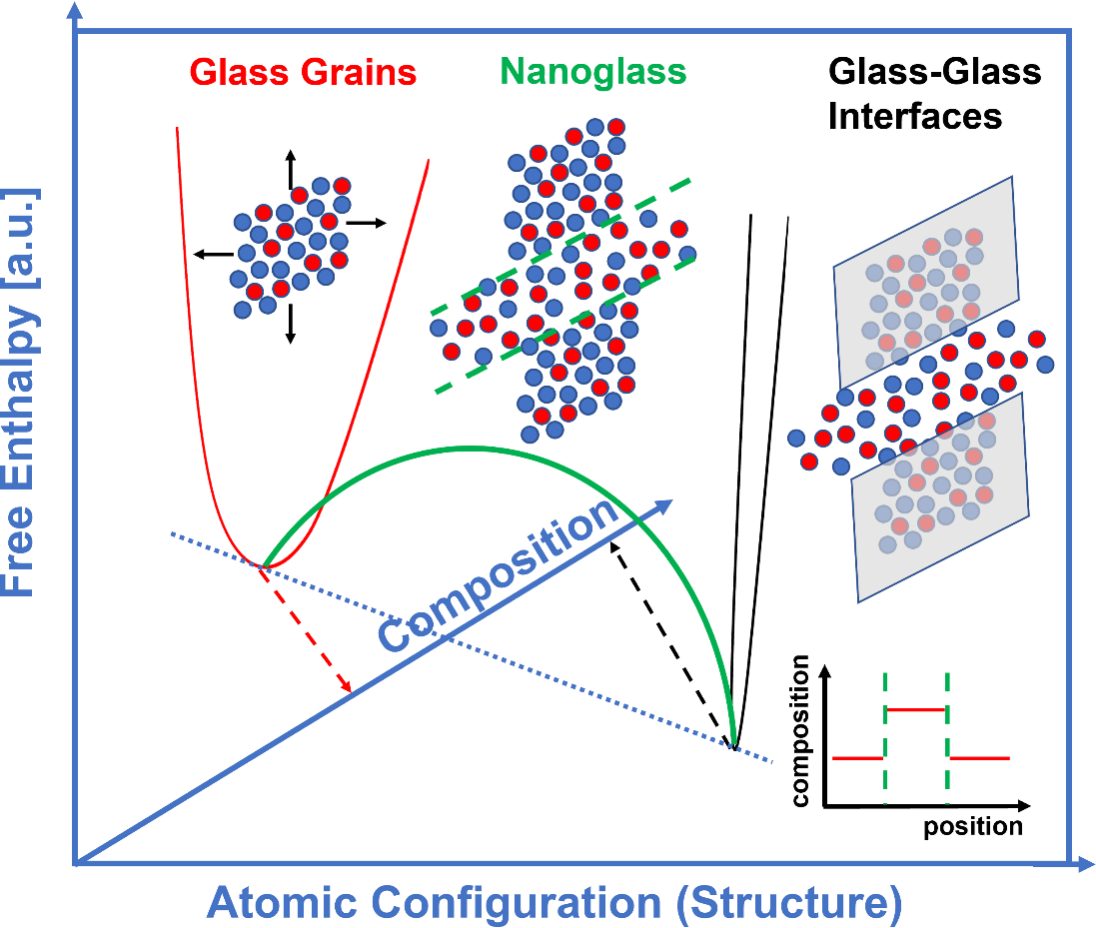
Schematic representation of the free enthalpy of a nanoglass (green curve) as well as its constituent phases, namely the glass grains (red curve) and the GGIs (black curve). The convex shape of the green curve stems from the excess energy contributions of the interfaces between glass grains and GGI phase. The linear common tangent (blue dotted line) would correspond to determining the free enthalpy of a classical two-phase state without taking curvature terms (due to interfaces) into account. The compositions of the glass grains and the GGIs differ due to the different chemical potentials of the two phases. This is indicated by the different amount of red or blue particles and also shown in the insert in the lower right corner, where the dashed green lines indicate the positions of the interfaces between glass grains and GGI phase. Both phases have also different local structures, as indicated by the different mean distances between particles and also by their different arrangements. The transparent grey boxes indicate that only the GGI phase contributes to the free enthalpy indicated by the black curve.

Nevertheless, the glass–glass interfaces might have a (significantly) decreased free enthalpy as compared to the glass grains. Their spatial extent justifies describing them as a distinct phase, in contrast to a description by a Gibbs’ excess as for internal crystallographic interfaces (i.e., relaxed grain boundaries) in crystalline materials [22], which have thicknesses, depending on the respective quantity, of one to two interatomic spacings, and where interface phases are often termed “complexions” [23]. Thus, based on classical thermodynamics of heterogeneous systems, the state of lowest free enthalpy of a nanoglass should be described by a “common tangent” construction involving both phases (Figure 2), glass grains and glass–glass interfaces, rather than by the lowest value of the excess free enthalpy of a (uniform) single phase (where the GGI phase, as a “conditional” phase, cannot form without the glass grains). At this point, it should be clarified that the GGIs are treated in this work as independent but “conditional” phase, always implying that this conditional phase can only exist as long as the abutting glass grains are present. At the same time, as glasses represent metastable or even out-of-equilibrium states, we treat the GGI similar to the glass grains as metastable, that is, we describe their well-relaxed states in this work. It should be noted that the linear common tangent construction for heterogeneous equilibria only applies to heterogeneous bulk systems where interface contributions to thermodynamic potentials can safely be neglected [24], as also pointed out already by Gibbs. Below, we discuss how the thermodynamic potentials of nanostructured systems with more than one co-existing phase, where interface contributions cannot be neglected, need to be described. This approach rationalizes the experimentally observed stability of the glass–glass interfaces as the lowest free enthalpy of the two-phase mixture is not determined by a linear common tangent but lies on a non-linear connecting curve at finite fractions of both phases.

The above description immediately triggers another question that pertains to the thickness of the GGI phase: Why do the glass–glass interfaces not grow in thickness until (almost) consuming the entire volume of a nanoglass? At this point, we need to consider the nanoscale structure of nanoglasses that inherently necessitates to take curvature contributions to thermodynamic potentials into account. In the realm of nanocrystalline matter, these curvature (or interface excess) contributions lead to size effects, for example, regarding the melting temperature of single-phase nanocrystals [25–27]. In the case of heterogeneous equilibria of nanostructured materials, that is, when two or more phases in a nanostructured system are (meta)stable under given boundary conditions, the excess contributions stemming from the interfaces between those co-existing phases need to be considered as well. Following our earlier work on the thermodynamic description of two-phase nanocrystalline systems [24], Figure 2 depicts schematically the thermodynamics of nanoglasses that are considered as nanocomposites of glass grains separated by the conditional glass–glass interface phase and with interfaces connecting the two phases. For simplicity, we summarize here excess free enthalpy contributions from the interfaces between glass grains and GGIs and strain contributions arising from the misfit at those interfaces into one term. Additionally, as we are discussing “final” states after sufficient relaxation, we assume for this conceptual treatment that the interfaces between GGIs and glass grains are sharp. For real-world nanoglasses, gradients of structure and/or composition might form, due to limited atomic mobility under the conditions of synthesis and storage as well as processing/analyses at not too high temperatures/extended annealing times. However, structural analyses show no indication of pronounced structural (or chemical) gradients [17]. Certainly, due to the nanoscale structures and projection artefacts in TEM-based methods, the presence or absence of gradients on length scales of 1–2 nm cannot be discussed rigorously and remains an experimental uncertainty. The situation is more complex concerning the possibility of having a gradient of structure between the GGIs and the glass grains as this question pertains to thermodynamics constraints as well as kinetics. As the excess free enthalpy contribution due to the interfaces between GGIs and glass grains depends on the total area of these interfaces and on the composition-dependent strain contributions, a linear common tangent construction does not describe the actual situation. Instead, the composition dependence of the interface excess leads to a convex free enthalpy functional, as also shown, for example, for two-phase equilibria in nanoscale particles when taking the heterophase interfaces into account [24]. As known from spinodal decomposition, only for single-phase states does a convex energy functional not describe equilibrium. For single-phase states, in fact, any convex free energy functional leads to spinodal-type decomposition due to thermodynamic instability. However, it should be realized that this does not hold for two-phase states where the common free energy functional of the two coexisting phases is given by a free energy functional that might have convex or concave shape, depending on the specific thermodynamics of the phases and their interactions. It is noted that the situation has analogues to the equilibrium between coherent phases, where the coherency strain energy is described by convex free energy functions similar to Figure 1 (see, for example, the work by Cahn and Larché [28]). Thus, while single-phase states with convex free energy functions would be unstable with respect to the formation of a two-phase state by spinodal decomposition, no such instability occurs here since two-phase states are considered from the outset for the present conditional equilibrium situation of the GGIs and the abutting glass grain phases.

Thus, the black convex curve in Figure 2 describes the total free enthalpy of the nanoglass consisting of glass grains and glass–glass interfaces. As shown in [24], the numerical solution of the governing equations leads to distinct values for the phase contents of both phases that depend on the nominal composition. In fact, a lever-rule-type linearization can only be applied if the interface excess terms are disregarded (i.e., for coarse-structured materials). These considerations are thus in line with the experimentally observed stability of a two-phase state consisting of glass grains and GGIs, each with their own specific structure and composition. Based on the above considerations it would be expected that for a given system the width of the GGI phase varies with nominal composition and that the local structure of the GGI phase remains constant. So far, no experimental observations concerning this expectation are reported, as a systematic variation of the composition of a nanoglass with fixed chemical constituents is absent.

In the same context, it has been suggested based on experimental measurements by Mössbauer spectroscopy that for a nanoglass of given composition, a specific and unique structure of the glass–glass interfaces forms [29–30]. This observation strengthens the above hypothesis that the glass–glass interfaces might present a conditionally “stable” glass state for a given composition, presenting a conditionally stable configuration at higher excess volume. At the same time, this experimental observation is also a strong argument against a purely kinetic stabilization of the GGIs. As the evidence for this effect is sparse, the following discussion on the kinetic stability of GGIs is regarded as a hypothesis. Based on the high amount of excess volume within the glass–glass interfaces, structural rearrangements are more viable as steric hindrances are reduced, giving rise to lower effective barrier heights between different initial configurations within the meta-basin that describes the states of the glass–glass interface phase. This situation favours the kinetic access of the system to a rather distinct and defined state of low free enthalpy. In other words, the meta-basin corresponding to the GGIs should be smoother and steeper, as compared to the meta-basins of conventional glasses, as also indicated schematically in Figure 2. This hypothesis is in line with the observations of long-time stability of the GGIs (even at increased self-diffusivity within the GGIs [31]) and the observed unique structure of glass–glass interfaces. Accordingly, the energy landscape of the glass–glass interface exhibits a distinct minimum in free enthalpy for a particular configuration, accompanied by comparatively low local barriers between states within the narrow meta-basin. While this favours the system’s evolution toward the global minimum, it simultaneously reduces the degree of structural variability accessible through relaxation, aging, or rejuvenation.

At constant nominal composition and after sufficient time for the GGIs to form a structure of low free enthalpy, property tuning of nanoglasses thus might involve relaxation, aging, or rejuvenation of the glass grains only, rendering the GGI phase rather an amorphous and not a vitreous phase, as discussed below in comparison to the case of amorphous, but not vitreous Ge.

Several open questions remain concerning the thermodynamic and kinetic stability of the glass–glass interfaces in nanoglasses. A fundamental aspect concerns the absence of nanoglass states in conventional glasses, or: Could nanoglasses form spontaneously during liquid quenching (or relaxation/rejuvenation)? This question entails also the question why nanoglasses have not been observed to form “accidentally”. In that context, it should be kept in mind that the excess volume, Δ*V*, within the glass–glass interfaces is of the order of 10% or higher, taking results from experimental analyses [7,31] and atomistic simulations [8] into account. In a crystalline material, the lattice would have become unstable at much lower values of Δ*V*. During classical vitrification via liquid quenching, the amount of volume change between the liquidus temperature of a material and its glass temperature amounts to about 1–3% at maximum. Thus, a condensed liquid phase might be too dense already, even at high temperatures above the liquidus temperature, to allow for the amount of excess volume required to form glass–glass interfaces.

At this point of the present discussion, we should remember that GGIs are described as structures in conditional thermodynamic equilibrium, that is, GGIs exist only in confinement between glass grains and not as isolated phases. Thus, the contributions of GGIs can only enter the thermodynamics of the system after glass grains and GGIs exist; spontaneous formation is ruled out. Additionally, the configurations of a conventional “bulk” glass are residing in the meta-basins corresponding to the glass grains, as schematically depicted in Figure 1. These states are separated from the meta-basin corresponding to the glass–glass interfaces by high barriers, and barrier-crossing is thus improbable. In order to form glass–glass interfaces inside a uniform bulk glass, excess volume would also need to be transported from the outside of the material to localized regions inside, involving long-range transport and thus requiring time scales that might be prohibitive, specifically with respect to the barrier heights along the transport pathway and concerning the competition with crystallization.

Based on the above hypothesis, it is possible to obtain a rational and consistent description of the thermodynamics of a nanoglass as a nanostructured conditional two-phase equilibrium. As indicated above, excess contributions stemming from the interfaces between glass grains and the glass–glass interface phase need to be considered and could play a pivotal role in describing and understanding nanoglasses. So far, the contribution of these interfaces has mostly been neglected.

When taking the definition of a “glass” as an amorphous solid that can be transferred to the liquid state via heating, thereby undergoing a continuous glass transition, at face value, it occurs that the glass–glass interfaces most probably do not fulfil this definition. Similar to, for example, amorphous germanium obtained by vapour condensation onto a cold substrate, a continuous transition from the amorphous state at low temperatures into a supercooled liquid at increased temperatures is impossible as a discontinuous change of the coordination number (for amorphous Ge) or the excess volume (for glass–glass interfaces) would be required [32]. As of now it remains unclear, how the structure of glass–glass interfaces changes at increased temperatures above the glass transition temperature of the respective bulk glass, due to the detrimental interference of crystallization. Nanoglasses thus most likely present vitreous–amorphous composites consisting of glass grains that are connected to an amorphous glass–glass interface phase through interfaces between them. Whether characteristic relaxation modes, also leading to the distinct glassy dynamics, occur also in the glass–glass interface phase remains a topic for future studies.

### Can amorphous materials have a “microstructure”?

At first glance, this question occurs as a contradiction in terms since “amorphous” means “shapeless”, which could be understood, in the realm of materials, as synonymous to “without structure”. However, due to the discrete nature of matter and the directional nature of interatomic (or intermolecular) interactions leading intrinsically to anisotropy, “structure” is omnipresent in any condensed state. For nanoglasses, it is the length scale of heterogeneity between the grains and the glass–glass interfaces that presents the characteristic feature. Adopting a view from the description of crystals, we could re-phrase the above question as: How can defects be introduced into an amorphous solid, and what is the nature of a defect in a glass? Neglecting trivial situations such as cracks or chemically distinct precipitates, similar questions have been followed already earlier, when discussing ion tracks in irradiated glasses [33–34] or shear bands in plastically deformed glasses [35–36]. These aspects and their possible connection to nanoglasses are elucidated below. Much earlier, classical work on dislocations has been initiated mostly for describing properties of the crystalline state. However, the conceptual derivation has started from describing elastic continua, that is, solids without any crystallography and thus without any “structure”. In fact, some models for plastic yielding of metallic glasses are based on disclination or dislocation approaches [37] that make use of the classical constructions defined by Volterra [38]. Thus, mesoscale (line-type) defects can be defined theoretically independent of the existence of a crystal lattice.

With nanoglasses, the glass–glass interfaces present the “defect” that is introduced to create a “microstructure” in the amorphous solid. As discussed above and shown in the literature, the term “interfaces” is somewhat misleading, as the thickness of these regions amounts typically to several nanometres, clearly exceeding the accepted definitions of an internal interface in a material. It thus seems more appropriate to describe nanoglasses as a composite consisting of two distinct amorphous phases that are, in turn, connected by interfaces. Similar to many glass composites synthesized via spinodal (or binodal) decomposition, both phases are amorphous and one phase is percolating. However, in the case of nanoglasses, both phases could have vast differences in excess volume, which is not necessarily the case in phase-separated glass composites. Therefore, nanoglasses bear more similarity with the concept of polyamorphism [39], where two glasses of identical composition but different MRO structure are present. So far, polyamorphism is well-known for polymers but has also been reported for materials with metallic bonding characteristics [40–41]. It should be emphasized that, in those cases, the differences in excess volume between the two amorphous phases are far lower as compared to nanoglasses. Nanoglasses thus present a new avenue for tailoring the properties of amorphous materials where the two-phase state with one amorphous phase residing in a remote meta-basin at high excess volume provides for unprecedented properties. Generally, any method that is capable of creating the “exotic” states of the glass–glass interfaces that distinguish this class of amorphous materials would present an acceptable pathway for nanoglass synthesis. Based on the above, it seems however that synthesis routes that are based on the homogeneous liquid as a starting state (as, for example, in melt-spinning, splat quenching, or mould-casting) will not allow for a pathway to nanoglass formation. Instead, cluster assembly, deposition routes, or more complex synthesis pathways that involve different processing steps in sequence are required and are described in the following section.

### Routes to nanoglass synthesis

Within the energy landscape approach, nanoglasses and conventional glasses occupy distinct meta-basins that, at temperatures below the glass transition, are separated by insurmountable energy barriers, thereby hindering uniform glasses from exploring the broader range of attainable states accessible to nanoglasses. These distinctive nanoglass states can be achieved through the utilization of processing pathways alternative to routes involving liquid quenching, which typically forms glasses that are uniform on length scales exceeding several tens of nanometres. In contrast, the majority of metallic nanoglasses have been produced through the consolidation of amorphous nanoparticles at elevated compaction pressure, enabling room-temperature processing [4–6], or through energetic deposition of amorphous clusters [42–43]. Consequently, when vitreous powder particles are compacted at low homologous temperatures, interfacial regions arise between the glass “grains”, characterized by increased excess volume. The increase of excess volume can be attributed to the nanometre-sized porosity among the consolidated glass nanoparticles, which facilitates localized dissipation of the excess volume, that is, initially condensed into pores and consequently results in alterations in the atomic density of the interfacial regions. Driving force for the dissipation of the condensed excess volume is given by the interfacial energy between the glass grains and vacuum (or the processing gas atmosphere), which disappears upon forming the glass–glass interface phase. For this process to occur, it is necessary that the interface excess free energy density of the interfaces between glass grains and glass–glass interface phase, together with the volumetric excess free enthalpy of the glass–glass interface phase, is lower than the excess free energy density of the free surfaces of the glass grains. The experimental observations of nanoglasses with considerable stability support this assumption.

In real-world nanoglasses, more than one chemical element constitutes the amorphous states. As discussed above and indicated in Figure 2, consolidation leads to the formation of glass–glass interfaces, resulting in a two-phase state of the nanoglass. As a consequence of fundamental thermodynamics, the chemical potentials of the constituents of the nanoglass thus are initially different in the two different amorphous phases and compositional re-distribution results to obtain a (conditionally) metastable state. Therefore, alloy compositions in the glass grains and in the GGI phase are necessarily expected to be different, as also experimentally confirmed. This situation stabilizes the two-phase state additionally concerning both thermodynamic and kinetic stability, similar to grain boundary segregation in polycrystalline materials.

Naturally, the compositional re-distribution between the two amorphous phases in nanoglasses needs to be considered when discussing property modifications and presents an additional tuning knob for property adjustment. It also presents a sink for impurities (e.g., oxygen or nitrogen), which might have affected some magnetic or elastic properties described in the literature. Thus, although the method of producing metallic nanoglasses through powder compaction subsequent to inert gas condensation initiated the field, similar to the early stage of research on nanostructured materials [44], it has proven overly intricate and lacking versatility in generating a diverse range of well-defined nanoglasses. Limited by the constraints elucidated in numerous studies, only a relatively small number of metallic nanoglasses have been successfully prepared, including PdSi, PdSiFe, FeSc, CuSc, FeCuSc, and CuZr [45]. Moreover, the fabrication of nanoglasses through inert gas condensation and compaction necessitates a sophisticated ultrahigh-vacuum-type apparatus. Consequently, only a select few research groups worldwide have endeavoured to synthesize metallic nanoglasses, substantially impeding progress in the field and restraining interest, despite promising improvements in mechanical and functional properties observed for nanoglasses. The emergence of alternative processing routes holds the potential to invigorate the development of this field and facilitate the comprehensive exploration of the full capabilities of nanoglasses.

In recent years, new synthesis routes for a nanoglass state have been suggested that are based on electrochemical methods [46–47] or deposition from the gas phase onto a substrate [48–51]. While electrochemical methods yield larger amounts of material in shorter times at reduced cost, when compared to deposition from the gas phase, inherent limitations concerning composition selection and purity exist. Often, suitable electrolytes for co-depositing glass-forming alloy compositions are lacking and/or the electrochemical potentials for co-depositing the different elements do not sufficiently overlap to generate the desired alloy composition. A versatile alternative is presented by depositing columnar thin-film nanoglasses from the gas phase onto substrates. Recently, the validity of this approach has been demonstrated for a Cu–Zr nanoglass that has been prepared by sputter deposition through columnar growth under conditions that avoid crystal formation [17,49,51].

**Figure 3:**
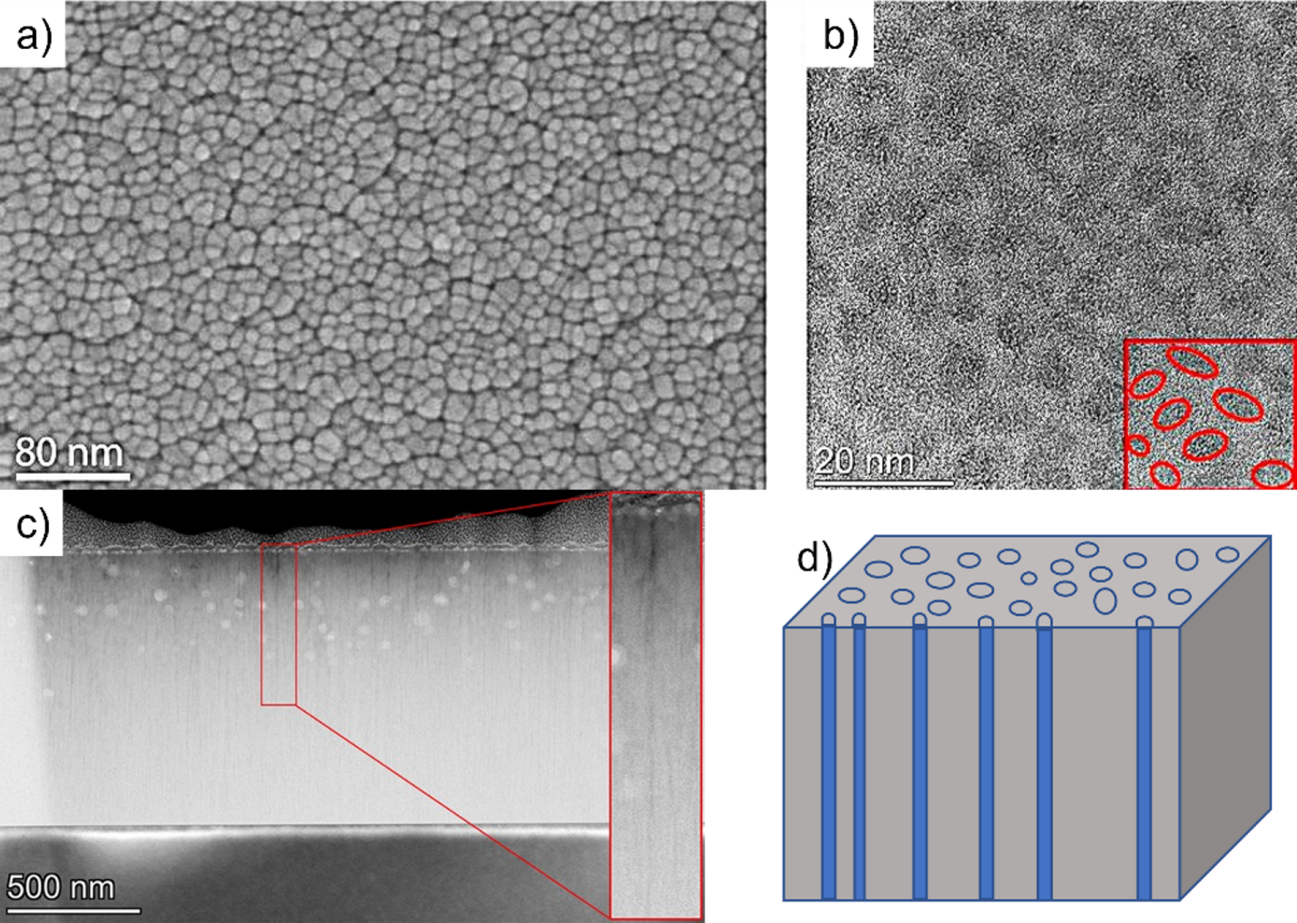
(a) SEM overview of the top surface of a columnar Cu–Zr nanoglass, showing the individual columns (i.e., the glass grains) connected by glass–glass interfaces. (b) HRTEM image of the same material as in (a), images along the plane-view direction with the incident electron beam parallel to the long axes of the nanoglass columns. The insert in red sketches the perimeters of the nanoglass columns. (c) HAADF-STEM image of the columnar nanoglass thin film imaged perpendicular to the direction of the long axes of the nanoglass columns. Note that in this imaging mode (i.e., *Z*-contrast imaging), brightness contrast corresponds to contrast in the density of the material. (d) Schematic illustration of the three-dimensional arrangement of glass “grains” (i.e., glass columns) indicated in blue inside the arrangement of columns that are connected by GGIs. Only few columns are indicated in blue to allow distinguishing them from the GGIs (grey). Figure 3a–c was adapted from [17] (©2023 H. Voigt et al., published by Wiley-VCH GmbH, distributed under the terms of the Creative Commons Attribution-NonCommercial-NoDerivatives 4.0 International License, https://creativecommons.org/licenses/by-nc-nd/4.0/). This content is not subject to CC BY 4.0.

Columnar thin-film nanoglasses have the additional advantage that the glass–glass interfaces can be aligned edge-on to a local probe, for example, a focused electron beam in a transmission electron microscope (TEM), so that projection artefacts that are inherent in the analysis of powder-compacted nanoglasses can be avoided. Figure 3a presents a scanning electron microscopy (SEM) overview of the columnar nanoglass thin films, highlighting their overall structural characteristics. A higher-resolution view is provided by TEM (Figure 3b), which reveals the nanostructured nature of the film. This structure comprises glass grains interspersed with channels, corresponding to glass–glass interfaces. An inset in the bottom right of Figure 3b emphasizes these different regions. Further confirmation of these distinct phases is provided by the cross-sectional high-angle annular dark-field scanning transmission electron microscopy (HAADF-STEM) image in Figure 3c, which differentiates the glass grains and the glass–glass interface regions based on contrasting dark and bright areas [17]. The channel widths, ranging from 2 to 4 nm, are notably smaller than the matrix regions. Enlarged views of individual channels are also presented as inset in Figure 3c, clearly illustrating the glass–glass interfaces. A schematic representation of the nanoglass structure is given in Figure 3d. These interfaces have been previously proposed in nanoglasses synthesized through alternative methods and have been confirmed for the first time for the case of columnar thin-film nanoglasses [17]. It is important to underscore that the observed grain-like structure in the sputtered films is not attributable to open porosity, as confirmed by TEM analyses and radiotracer diffusion measurements. In contrast, nanoglasses fabricated through inert gas condensation frequently exhibit substantial porosity, which can compromise their structural integrity. This suggests that sputtered films may offer a more versatile and more reliable approach for nanoglass fabrication, potentially overcoming the porosity- and impurity-related limitations of nanoglasses prepared by inert gas condensation.

Tracer diffusion measurements were conducted using ion beam sputtering and time-of-flight secondary ion mass spectrometry (ToF-SIMS) on columnar Cu–Zr nanoglass [31]. The results revealed significantly enhanced diffusivities of the glass–glass interfaces in nanoglasses compared to their homogeneous amorphous counterparts. Specifically, diffusivities in the columnar thin-film Cu–Zr nanoglass samples were consistently higher in the temperature range of 573 to 673 K. Furthermore, a quantitative assessment of the excess volume in the glass–glass interfaces of this nanoglass relative to chemically similar homogeneous glasses indicated an approximate 18% increase in excess volume. As described above, this increase exceeds by far typical relaxation-induced density variations (which remain in the range of less than 2%) and aligns well with previous findings on columnar thin-film nanoglass samples obtained via TEM-based methods [17]. When the tracer diffusion data is analysed in analogy to grain boundary diffusion in polycrystalline materials, with the glass–glass interfaces presenting the fast and the glass grains presenting the slow diffusion pathways, a diffusion acceleration by about four orders of magnitude of the diffusion along the glass–glass interfaces compared to diffusion in the homogeneous glass of identical nominal composition is obtained [31]. In fact, the values for the diffusivity enhancement through the glass–glass interface phase are numerically about similar to diffusivity values obtained for diffusion along shear bands in deformed bulk metallic glasses that also show a strong acceleration of diffusion in comparison to the homogeneous glass [52–53].

Such high diffusivities could stimulate a concern about the stability of the nanoglass state, that is, concerning the stability of the glass–glass interfaces. However, in addition to the discussion on the thermodynamic aspects above, the situation resembles the discussion concerning the stability of so-called “non-equilibrium” grain boundaries in severely deformed metals and alloys [54–55]. Those defects are also observed over extended durations, even under thermal conditions that allow for significant diffusion distances, while presenting specific grain boundary diffusivities that are enhanced by several (six to eight) orders of magnitude with respect to the average grain boundary diffusivity along relaxed high-angle grain boundaries in the chemically identical material. In that case it has been suggested that the distribution of the excess volume along the non-equilibrium grain boundaries, giving rise to the enhanced diffusivity, should not be uniform. Thus, patches of low excess volume would provide for stability (in analogy to a “lock-in” mode [56]), while interconnected regions with an increased excess volume provide for high atomic mobility. For the glass–glass interfaces in nanoglasses, high diffusivities along the interface phase together with low perpendicular mobility could occur due to kinetic constraints stemming from compositional and structural differences of the two glass phases. Additionally, a thermodynamic contribution due to a conditional two-phase equilibrium as described above and depicted schematically in Figure 2 might reduce the driving force for dissolving one of the amorphous phases (i.e., grains and GGIs) into each other. Thus, atomic jumps between the glass grains and the glass–glass interfaces would be less probable as jumps within the glass–glass interfaces due to the energy landscape particulars described above.

To explore also the relaxation kinetics in nanoglasses, annealing times during diffusion measurements were also varied [31]. The results from diffusion measurements and TEM-based analyses [57] demonstrated that neither the nanoglass nor the homogeneous glass samples were fully relaxed, particularly at the lower temperatures studied. Consequently, diffusivity decreased with longer annealing times. This annealing-time-dependent decrease in diffusivity, despite the pronounced excess volume observed in nanoglasses, suggests that a detailed examination of local structural configurations and bonding characteristics and their dependence on (relaxation) time is necessary, but still lacking, to fully understand the underlying mechanisms. The results on atomic diffusion, together with the direct structural observations, confirm that columnar thin-film nanoglasses are “true” nanoglasses in the exact sense of the original definition. Moreover, the columnar geometry allowed for imaging and structural analyses of the glass–glass interfaces for the first time. The diffusion measurements verified that the atomic transport through the glass–glass interface phase proceeds significantly faster than in the bulk phase of the chemically identical homogeneous glass.

With the recent verification that thin-film deposition methods also allow for synthesizing nanoglasses, a versatile approach is now available to design glasses with a “microstructure” consisting of a characteristic intrinsic length scale and tuneable fractions of coexisting glass states without the previous, synthesis-related restrictions. The inherent limitation of deposition methods to produce thin films does not appear as a strong limitation since these materials are important for fundamental research or for applications where no bulk material is required (see below). With this new opportunity for synthesizing nanoglasses with tailored structures and tuneable properties, applications in areas such as sensing, catalysis, energy harvesting, or surface protection are in perspective, where the enhanced atomic mobility in the glass–glass interfaces is of specific benefit for the underlying transport processes. Moreover, important fundamental questions concerning the glass state and concerning the range of properties accessible to glasses can now be tackled and need to be addressed to establish the structure–property correlations for this new class of amorphous composite materials.

### Are glass–glass interfaces, shear bands, and ion tracks related?

As indicated above, similarities might exist between glass–glass interfaces and structural heterogeneities in metallic glasses that have been processed by plastic (shear) deformation or, alternatively, that have been irradiated by ions with a high kinetic energy. Plastic shear deformation leads to extended plate-like shear bands [57], and ion irradiation causes rod-like ion tracks [33]. The thickness of the shear bands and the diameter of the ion tracks are in the range of few nanometres, quite comparable to the width of glass–glass interfaces in nanoglasses. Interestingly, the ranges of excess volume increase and diffusivity increase of the GGIs, as compared to their chemically identical homogeneous glass counterparts, are very similar to what has recently been reported for shear bands in plastically shear-deformed bulk metallic glasses [52,58–59]. In both cases, direct structure-sensitive measurements (i.e., TEM-based analyses) as well as indirect but highly sensitive measurements such as radiotracer diffusion indicate a spectacular amount of excess volume locally stored in the glass–glass interfaces as well as in shear bands.

In fact, for an Al-based metallic glass, values of the local excess volume up to 8% compared to the uniform metallic glass of identical composition have been determined [58,60]. Different to nanoglasses, the excess volume in shear bands shows a spatial modulation along the shear band with a characteristic modulation distance of the order of about 200 nm, independent of the composition of the metallic glass and with alternating regions of decreased or increased atomic density along the shear bands [58,60–61]. The origin of the specific modulation distance is as yet unresolved. Yet, the observation of very similar modulation distances for plastically deformed metallic glasses of rather different chemical composition and different characteristics, such as fragility or glass-forming ability, leads to the hypothesis that the modulation distance is an intrinsic consequence of the glass structure and the deformation processing conditions. Recently, a model based on continuum theory has been advanced that is able to link the magnitudes of density alterations along the shear band to the elastic properties of the glass [61]. Yet, the “wavelength” of the spatial density modulations remains as yet unexplained.

Radiotracer diffusion measurements on plastically shear-deformed bulk metallic glasses containing shear bands have shown that the enhanced excess volume (possible in conjunction with its non-uniform distribution) leads also to remarkably enhanced diffusivities along the shear bands [52]. In fact, an enhancement by six to eight orders of magnitude even for partially relaxed states has been determined experimentally [62]. Observations by atom probe tomography independently indicated strongly enhanced atomic transport kinetics [63]. It is important to note also in this context that the fast diffusion pathways have extended life times, even under conditions that allow for significant diffusion distances exceeding the width of the shear bands by orders of magnitude. Based on the definition of a nanoglass and the comparison of the characteristics of structure and atomic kinetics, deformation-induced shear bands in metallic glasses and glass–glass interfaces in nanoglasses show remarkable similarities. Thus, shear straining exceeding the elastic limit can offer an opportunity for nanoglass formation of larger volumes, that is, bulk samples, given that the material can be vitrified as a bulk metallic glass during an initial quenching step. While constitutionally more strongly confined than vapour deposition, this route enables nanoglass formation at reduced cost.

This triggers the question whether mechanical straining would allow for synthesizing an amorphous material that consists entirely of a “shear band phase”? In this context, bulk metallic glass samples have been severely deformed by applying extremely high levels of torsional strain under a high hydrostatic pressure via so-called “high-pressure torsion” [64–66]. The resulting material in fact shows mechanical and thermodynamic characteristics of a very high fraction of the shear-band phase. Yet, a complete transformation of the entire volume of the homogeneous glass to the shear band phase could not been attained, even after applying excessive amounts of strain. In view of the above discussion on the thermodynamics of nanoglasses as two-phase systems, the retention of glass grains might be necessary and dictated by thermodynamics. This hypothesis suggests that shear bands in plastically deformed metallic glasses and glass–glass interfaces in columnar thin-film nanoglasses bear important and strong similarity.

Naturally, the origin of this apparent similarity is of high scientific interest. So far, we can only speculate as not enough is known concerning the detailed local structures, the MRO motifs and their distributions in different glasses and nanoglasses, or their dependence on processing pathways and relaxation, aging, or rejuvenation. Yet both shear bands and glass–glass interfaces were formed under non-equilibrium conditions involving non-liquid states or athermal excursions within the energy landscape, such as through plastic shearing. While a deposition from the gas phase involves highly excited states and thus allows for far-reaching excursions in the energy landscape, plastic shearing causes tilting of the energy landscape in addition to elevating the free enthalpy of the deformed glass. Thus, both synthesis and processing pathways favour excursions into new meta-basins. Within this view, it seems that glasses enable the formation of high-excess-volume configurations if the states can escape from the confinement of the meta-basins defined by the liquid as the starting state of glass formation.

This thought leads also to the comparison of glass–glass interfaces with ion tracks in glasses after ion irradiation. Recent experimental analysis on bulk metallic PdNiP glasses after irradiation with different swift heavy ions (SHIs) at different fluences shows clearly the formation of ion tracks and the concomitant swelling of the irradiated glass, indicating the increase of excess volume [34]. Low-temperature heat capacity measurements substantiated an irradiation-induced increase of the boson peak height with increasing fluences, similar to severe plastic deformation. Diffusion measurements using a radioactive Ag isotope as tracer also revealed increased diffusion rates in the irradiated samples. Nanoindentation measurements show enhanced plasticity and a strong decrease or even absence of shear localization in the ion-irradiated glass, which was correlated with an increased heterogeneity of the MRO as indicated by variable-resolution fluctuation electron microscopy. Additionally, an extremely large enhancement of the relaxation enthalpy stored in the material has been measured by differential scanning calorimetry. The derived data substantiates a prominent enhancement of the excess volume in the ion tracks. However, the magnitude of the SHI-induced effects is much smaller as compared to the modifications of the excess volume or the diffusivity in shear bands or in glass–glass interfaces. For the material within the ion tracks, a relative increase of the excess volume by about 30% was estimated based on the measured diffusivities. This value needs to be compared to the absolute increase of the excess volume by about 10% in shear bands or glass–glass interfaces. Correspondingly, the diffusivity of the ion tracks showed an increase by only one to two orders of magnitude compared to the homogeneous glass of identical composition. As indicated above, the same comparison for shear bands or glass–glass interfaces yields an increase by about four to eight orders of magnitude.

Following the above discussion, the relatively lower magnitude of modifications by SHI might be related to the absence of processing steps that allow the state of the glass to escape from the meta-basin defined by the initial liquid state. During SHI irradiation, the deposition of the kinetic energy of the ions leads to a local, very rapid and strong temperature excursion. The material inside the ion tracks thus transitions to the liquid state and, due to the coupling to the glass environment, subsequently cools extremely fast. Thus, the material inside the ion tracks is extremely rejuvenated, as it was vitrified at an ultrafast quenching rate. However, the above comparison indicates that, as the origin of the glass is the liquid state, it cannot reach the meta-basin of the glass–glass interfacial phase. The results also indicate that the residual stresses generated during the processes of rapid melting and vitrification of the ion tracks are not sufficient to enable the escape from that meta-basin.

The comparison with shear bands or ion tracks indicates that not all glasses with a heterogeneous structure might qualify as nanoglasses. The observations on nanoglasses suggest that the formation of glass–glass interfacial states, or structurally analogous configurations, requires processing pathways that access athermal excursions or highly excited states. The currently available results indicate that such states cannot be achieved through thermal treatments of a melt-quenched (i.e., “vitrified”) glass only, including rapid thermal excursions such as during ion irradiation. However, the comparison between glass–glass interfaces and shear bands in plastically shear-deformed glasses indicates the potential for processing pathway tuning to obtain nanoglasses for various compositions and also in quantities exceeding the capabilities of thin-film deposition or inert gas condensation methods.

### Potential for property tuning of nanoglasses

**Figure 4:**
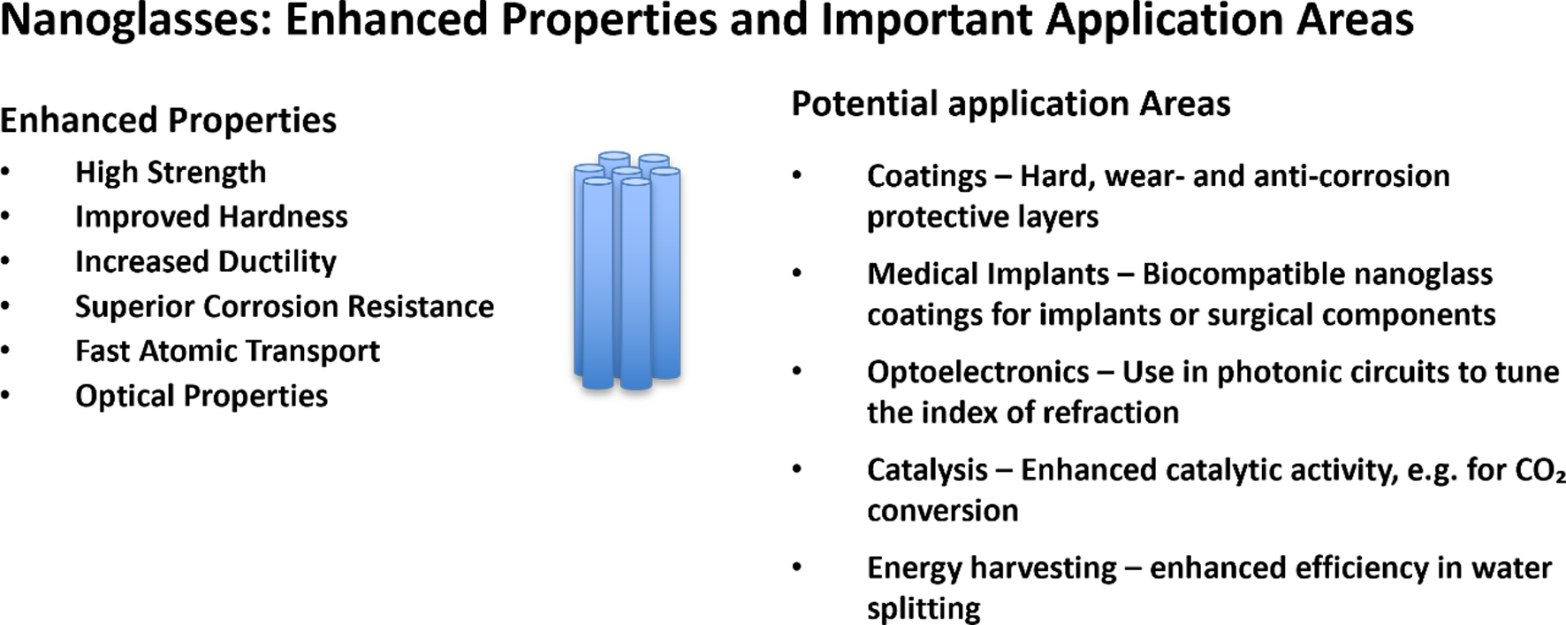
Property enhancements of nanoglasses and potential application areas of columnar thin-film nanoglasses.

Classical nanoglasses based on powder compaction and, in fewer cases, columnar nanoglasses have already been reported to present a broad spectrum of properties and property combinations that make them attractive novel materials for a broad range of applications, mostly in areas where nanoglass thin films or coatings are required (Figure 4). The interested reader is directed to recent reviews on the status of the field [45]. The following section is thus devoted to give a short outlook of potential application directions of columnar thin-film nanoglasses. Concerning property enhancement, nanoglasses have been reported to exhibit higher hardness and yield strength as well as higher ductility than conventional glasses due to the obstruction of shear band propagation by GGIs [67]. Whether this strengthening is related to the presence of structural differences alone (i.e., different amounts of excess volume coupled with different MRO structure) or to the intrinsic compositional differences between glass grains and GGI in multicomponent nanoglasses remains unclear. Tailoring the density and chemistry of interfaces might enable the tuning of plastic deformation pathways and promote enhanced plastic deformability without compromising strength, which would also allow for improving a critical basic property of almost any material concerning applications.

Magnetic nanoglasses demonstrate enhanced soft magnetic properties due to localized atomic rearrangements at GGIs that reduce magnetic anisotropy and domain wall pinning [68]. Nanoglasses have also been reported to provide excellent properties in the area of energy materials, that is, for catalysis [47], ionic transport, as well as water splitting. In oxide-based nanoglasses, the high excess volume at GGIs accelerates ionic diffusion, making them attractive for solid-state batteries and fuel cells [69]. Moreover, catalytic activity could be tuned by introducing active species at GGIs, where enhanced surface area and undercoordinated sites act as reaction centres. Similar approaches might also be effective in tuning biomedical properties such as cell growth on nanoglass surfaces, which could promote applications in prosthetics. While the structural length scale of nanoglasses seems too short for effectively coupling to cell adherence, the accelerated material transport through the GGI could serve as a network of fast transport channels that might transport atomic or molecular species to or away from the nanoglass/cell interface to stimulate faster cell growth and/or stronger adherence.

In the area of materials for optical applications, glasses have always occupied a prominent position. In addition to the wide electronic bandgap of optical, that is, mostly oxide-based glasses, the opportunity to tune the refractive index through annealing below the glass transition temperature is the base for their widespread use. Nanoglasses, as discussed above, have a strongly enhanced potential for more significant tuning of their optical properties due to their enhanced capability for excess volume adjustment. As the length scale of spatial heterogeneity amounts to tens of nanometres, interactions with light in the visible spectrum should not be strongly affected by enhanced scattering. Also, carefully controlled heterogeneity and tuning of photoluminescence might offer new potential applications in photonic devices.

Nanoglasses have emerged as promising candidates for enhancing the efficiency of water splitting processes [69–72]. Water splitting, which involves breaking water molecules (H_2_O) into hydrogen (H_2_) and oxygen (O_2_) using energy (usually electricity or sunlight), is a key step for producing clean hydrogen fuel. Traditional catalysts, often based on metals or semiconductors, can be inefficient, expensive, or environmentally harmful. Nanoglasses, due to their unique nanostructural properties, offer several advantages in water splitting. Their high surface area, chemical stability, and ability to enhance charge carrier mobility make them excellent candidates for use as catalysts or catalyst supports. The glassy structure can also be tailored to optimize the interaction with water molecules, leading to improved reaction kinetics. Moreover, nanoglasses can be engineered to enhance light absorption and reduce recombination of charge carriers, thus increasing the overall efficiency of the photoelectrochemical water splitting process. By integrating nanoglasses into the water splitting systems, researchers are developing more sustainable, cost-effective, and high-performance materials that could significantly improve hydrogen production from water.

Nanoglasses are currently considered as next-generation functional materials. Unlike traditional metallic or oxide glasses, nanoglasses exhibit a unique interplay of structural heterogeneity, high excess volume regions, and tuneable interfaces, which can be leveraged to enhance mechanical, magnetic, optical, and catalytic properties. Several strategies have emerged to exploit the unique structural characteristics of nanoglasses for functional improvements that rely on the presence of GGIs. As of now, most of the potential properties and property enhancements of nanoglasses, as well as their structural origins, wait to be explored in detail. Similarly, the use of dopants or alloying elements selectively segregated at GGIs can potentially enhance target properties and can be utilized for site-selective energy band tuning. These directions of research are mostly open for future investigations.

The widespread study and use of nanoglasses have been hampered/hindered for years by the complexity of the gas phase synthesis route using compaction of highly reactive nanoparticles. The fact that it has been shown that glass–glass interfaces with comparable structure and properties can also be created using thin-film techniques extensively available in many laboratories around the world can provide a new push towards innovative research leading potentially to applications.

## Conclusion

Nanoglasses, particularly in the form of columnar structures (or morphology) synthesized through deposition processes, represent a significant advancement in the field of amorphous materials, where the precise manipulation of nanoscale heterogeneity offers a versatile platform for enhancing functional properties. Descriptions should consider nanoglasses as inherent composites consisting of glass grains and glass–glass interface phases that are connected by interfaces between them. By leveraging controlled interface engineering and microstructural design, nanoglasses have the potential to surpass traditional glasses in mechanical, magnetic, catalytic, and electronic applications. Furthermore, they provide a broader range of tunability for property modification compared to crystalline materials. Ongoing interdisciplinary research, integrating materials synthesis, characterization, and computational modelling, will be crucial for unlocking the full potential of nanoglasses. This research will deepen our understanding of the structure–property relationships in glasses and drive innovative technological breakthroughs.

## Data Availability

Data sharing is not applicable as no new data was generated or analyzed in this study.
